# Association of the lipoprotein receptor-related protein 2 gene with gout and non-additive interaction with alcohol consumption

**DOI:** 10.1186/ar4366

**Published:** 2013-11-04

**Authors:** Humaira Rasheed, Amanda Phipps-Green, Ruth Topless, Jade E Hollis-Moffatt, Jennie Harré Hindmarsh, Christopher Franklin, Nicola Dalbeth, Peter B Jones, Douglas HN White, Lisa K Stamp, Tony R Merriman

**Affiliations:** 1 Department of Biochemistry, University of Otago, Dunedin, New Zealand; 2 Ngati Porou Hauora Charitable Trust, Te Puia Springs, New Zealand; 3 Department of Medicine, University of Auckland, Auckland, New Zealand; 4 Department of Rheumatology, Waikato Hospital, Hamilton, New Zealand; 5 Department of Medicine, University of Otago, Christchurch, New Zealand

## Abstract

**Introduction:**

The T allele of a single nucleotide polymorphism (SNP: rs2544390) in lipoprotein receptor-related protein 2 (*LRP2*) is associated with higher serum urate and risk of gout in Japanese individuals. SNP rs2544390 also interacts with alcohol consumption in determining hyperuricemia in this population. We investigated the association of rs2544390 with gout, and interaction with all types of alcohol consumption in European and New Zealand (NZ) Māori and Pacific subjects, and a Māori study cohort from the East Coast region of NZ’s North Island.

**Methods:**

Rs2544390 was genotyped by Taqman®. From NZ a total of 1205 controls and 1431 gout cases clinically ascertained were used. Publicly available genotype and serum urate data were utilized from the Atherosclerosis Risk in Communities (ARIC) study and the Framingham Heart Study (FHS). Alcohol consumption data were obtained by consumption frequency questions in all study cohorts. Multivariate adjusted logistic regression was done using STATA.

**Results:**

The T allele of rs2544390 was associated with increased risk of gout in the combined Māori and Pacific Island cohort (OR = 1.20, *P* = 0.009), and associated with gout in the European subjects, but with a protective effect (OR = 0.79, *P*_Unadjusted_ = 0.02). Alcohol consumption was positively associated with risk of gout in Māori and Pacific subjects (0.2% increased risk/g/week, *P* = 0.004). There was a non-additive interaction between any alcohol intake and the risk of gout in the combined Māori and Pacific cohorts (*P*_Interaction_ = 0.001), where any alcohol intake was associated with a 4.18-fold increased risk in the CC genotype group (*P* = 6.6x10^-5^), compared with a 1.14-fold increased risk in the CT/TT genotype group (*P* = 0.40). These effects were not observed in European subjects.

**Conclusions:**

Association of the T-allele with gout risk in the Māori and Pacific subjects was consistent with this allele increasing serum urate in Japanese individuals. The non-additive interaction in the Māori and Pacific subjects showed that alcohol consumption over-rides any protective effect conferred by the CC genotype. Further exploration of the mechanism underlying this interaction should generate new understanding of the biological role of alcohol in gout, in addition to strengthening the evidence base for reduction of alcohol consumption in the management of gout.

## Introduction

Elevated serum urate in humans is the central risk factor for gout. To date genome-wide association scanning has identified 28 loci that explain approximately 6% of the variation in serum urate levels in European Caucasians [1]. Prominent associations are with renal (*SLC2A9, SLC17A1, SLC22A11, SLC22A12, PDZK1*) and gut (*ABCG2*) molecules directly involved in urate transport. Not unexpectedly some of these genes are also associated with gout [1-4]. In Japanese the T allele of single nucleotide polymorphism (SNP) *rs2544390* in the *LRP2* gene has been associated with elevated serum urate by genome-wide scanning [5] and is associated with gout in Japanese, conferring a moderate risk of odds ratio (OR) = 1.32 [6]. LRP2, also known as megalin, is a member of the lipoprotein receptor-related protein (LRP) family, with its predominant function in lipid metabolism through controlling the activity of lipoprotein lipase [7,8]. LRP2 plays a role in the reabsorption and metabolism of renal glomerular-filtered substances, including albumin and low molecular weight proteins [9].

Along with inherited genetic variants, dietary factors, such as sugar-sweetened beverages and alcohol consumption influence serum urate levels and gout risk [10-13]. Beer and spirits (but not wine) intake is positively correlated with serum urate and the relative risk of gout increases with increasing alcohol (particularly beer) consumption in men [10,14]. Recently, in a Japanese male sample set there was evidence for a *LRP2*-environment interaction, where there was a significantly increased risk conferred by the rs2544390 TT genotype for hyperuricemia with increased alcohol consumption [15].

This study aimed to test for an association of *LRP2 rs2544390* with gout and serum urate in European Caucasian and Eastern and Western Polynesian ancestral groups of New Zealand. The interaction of alcohol consumption with genotype was also evaluated.

## Methods

### Study participants

The New Zealand subjects consisted of gout cases clinically ascertained by the American College of Rheumatology criteria [16]. The comparison group was self-reported in their lack of a diagnosis of gouty arthritis. Recruitment was during the period 2006 to 2012. All variables except for biochemical measurements and body mass index (BMI) were self-reported. Demographic and clinical data are reported in Additional file 1: Table S1. For initial genetic analysis subjects were divided into four ancestral groups [2]; European Caucasian (555 cases, 282 controls), Eastern Polynesian (EP; Cook Island and NZ Māori; 374 cases, 533 controls), Western Polynesian (WP; Samoa, Tonga, Niue, Tuvalu and Tokelau; 285 cases, 165 controls) and mixed Eastern and Western Polynesian (EP/WP; 21 cases, 18 controls). A separate Māori sample set from the *rohe* (area) of the Ngati Porou *iwi* (tribe) from the Tairawhiti region on the East Coast of the North Island of New Zealand was included, ascertained as described above (196 cases, 207 controls). This sample set was recruited in collaboration with Ngati Porou Hauora (health service) (NPH). The New Zealand Multi-Region Ethics Committee (MEC/105/10/130) granted ethical approval, with the Northern Y Region Health Research Ethics Committee granting ethical approval for the Ngati Porou Hauora study (NTY07/07/074). All participants gave fully informed written consent for taking part in the study.

Data from the Framingham Heart Study (FHS) (Generation 3 only) and Atherosclerosis Risk in Communities (ARIC) cohorts were also used for evaluating associations with serum urate in European Caucasians. The approval number was #834 for accessing data from the ARIC and FHS studies under the project name ‘The Genetic Basis of Gout.’ Subjects from the ARIC and FHS studies who self-reported as taking diuretic medication, who were first degree related and who were not of European Caucasian ancestry were excluded. The ARIC dataset consisted of 4,144 individuals and the FHS of 3,047 individuals.

### Data collection

Serum urate levels were measured for NZ subjects by the uricase oxidation method, the end point determined by a Roche chemistry modular P/D analyzer. In all data sets, alcohol consumption was obtained by food frequency questionnaire. NZ participants were asked at recruitment how many servings of beer, spirits, wine and other alcohol were consumed in the previous week. For conversion of servings/week intake into g/week intake, values were multiplied by the estimated quantities of alcohol in each category of drink (beer = 10 g/300 ml serving; wine = 15 g/200 ml serving; spirits = 10 g/serving; other = 10 g/serving). For ARIC, alcohol data were supplied as g/day (from examination 1 in the years 1987 to 1989) usage and converted into g/week for this study. For FHS, alcohol intake data were obtained as number of servings per week (beer, wine and liquors) (from visit 1 for Generation 3 in the years 2002 to 2005) and converted into g/week alcohol as described above. Serum urate data were obtained from examination 1 for ARIC and visit 1 for FHS.

### Sample preparation and genotyping

Separated white blood cells were used for DNA preparation by the guanidine hydrogen chloride method with chloroform extraction [17]. Taqman® genotyping for *rs2544390* was performed using a Lightcycler® 480 Real-Time Polymerase Chain Reaction System (Roche Applied Science, Indianapolis, IN, USA) in 384 well plates. The FHS cohort had been genotyped by the Affymetrix SNP 5 platform and a custom designed gene-centric 50 K SNP platform and *rs2544390* genotype was imputed using MACH1 v1.0.15 with the HapMap2 CEU sample set as reference haplotypes. In the ARIC sample set *rs2544390* had been genotyped on the Affymetrix SNP 6 platform.

When comparing with the data of Hamajima *et al*. [15], it was important to be certain about allele assignments at *rs2544390 -* uncertainties can arise when allele prevalences are approximately 50% as is the case in the Japanese population. Our Taqman-generated genotypes were 100% concordant with genotype data generated by the Affymetrix Axiom genotyping platform in a subset of 66 NZ samples. Furthermore, the C allele is the major allele in European Caucasian [18], consistent with our Taqman genotype data. We confirmed consistency in allele assignment between our and the Hamajima *et al*. [15] data by genotyping using Taqman 15 samples (5 CC, 5 CT, 5 TT) from the Hamajima *et al*. study.

### Statistical analysis

Regression analysis was used to investigate relationships between the variables in this study. For continuous response variables, this involved standard linear regression. For binary response variables (that is, discrete variables with only two levels), logistic regression was used. Both main effects and interaction terms were included in the models where appropriate. The adjusted OR for a single explanatory variable was obtained by using the logistic regression coefficient. An adjusted OR for the same variable was obtained by including additional variables in the logistic regression model, again taking the value of the model coefficient relating to the variable in question. Logistic regression analysis was used to assess the association between gout (binary response variable) and alcohol consumption and *LRP2* genotype (explanatory variables), and linear regression analysis was used to assess the association between serum urate levels (response variable), and alcohol consumption and *LRP2* genotype (explanatory variables). Unstandardized regression coefficients are reported. Main effects were included in the model for alcohol consumption and *LRP2*, and for the covariates age, sex, BMI and sugar-sweetened beverage consumption. Individuals with missing data from any variable were excluded from analysis. An interaction term between alcohol consumption (both as a continuous and a binary variable) and *rs2544390* genotype (binary: those who had at least one T allele (T/C or T/T) and those with none (C/C)) was also included. All analysis was done using Intercooled STATA™ software version 8.0 (College Station, TX 77845, USA). Meta-analysis of allele counts was performed in R within STATA™ using rmeta [19] to calculate combined ORs and to evaluate heterogeneity between studies. Coefficients with *P* ≤0.05 were considered to indicate a nominally significant association between the response and explanatory variables.

## Results

**Figure 1:**
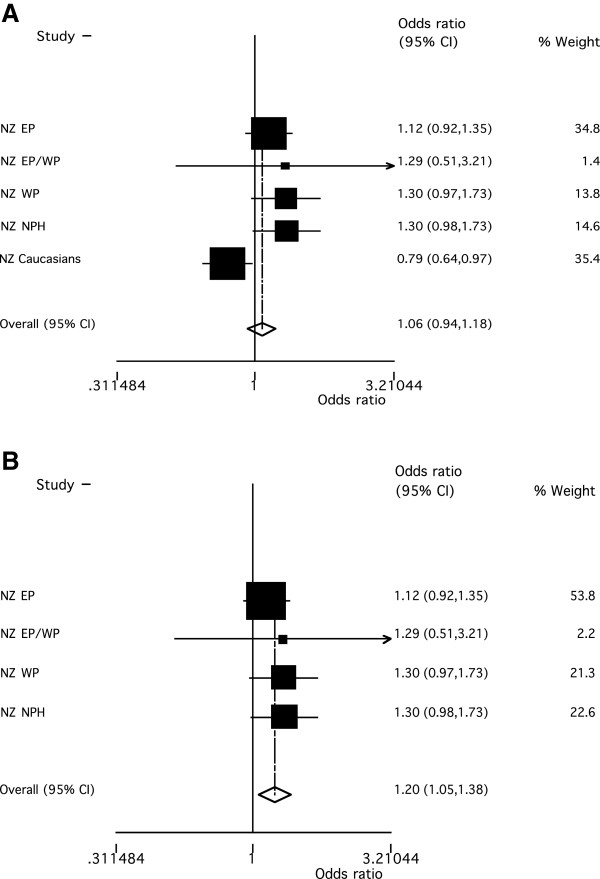
**Meta-analysis of association of *rs2544390 *with gout. A)** with gout in all study groups (*P*_Het_ = 0.015 and *P*_OR_ = 0.36), **B)** with gout in NZ Māori and Pacific Island groups (*P*_Het_ = 0.774 and *P*_OR_ = 0.009).

In the individual sample sets there was evidence for association of *rs2544390* with gout in the European Caucasian sample set (Table 1; OR_Unadjusted_ = 0.79, *P* = 0.02; OR_Adjusted_ = 0.73, *P* = 0.07). However, there was no evidence for association when all groups were combined in meta-analysis (Figure 1A; OR = 1.06, *P* = 0.36) although the *P*_Het_ = 0.015 in the meta-analysis suggested some heterogeneity. A potential source of heterogeneity was ancestry; therefore, groups were combined by meta-analysis in ancestral-specific groups. This analysis revealed the T allele to be associated with increased gout risk in people of Māori and Pacific ancestry (Figure 1B; OR = 1.20, *P* = 0.009) with, in contrast to the effect in European Caucasian, the minor allele conferring risk (Table 1; Figure 1A). There was no evidence for an association of *rs2544390* with serum urate by linear regression testing in non-gout (including ARIC and FHS) sample sets (Table 2).

**Table 1:** **Association analysis of *rs2544390 *** **with gout **

**Sample set**	**Case genotypes, number (frequency)**				**Control genotypes, number(frequency)**				**Unadjusted**		**Adjusted**	
	**CC**	**CT**	**TT**	**T frequency**	**CC**	**CT**	**TT**	**T frequency**	**Allelic OR, (95% CI)**	***P***	**Allelic OR,(95% CI)**	***P***
European Caucasian	206(0.371)	272(0.490)	77(0.139)	426 (0.384)	95(0.337)	125(0.443)	62(0.220)	249 (0.441)	0.79 (0.64-0.97)	0.02	0.73 (0.52-1.03)	0.07
Eastern Polynesian	63(0.168)	166(0.444)	145(0.388)	456 (0.610)	94(0.176)	257(0.482)	182(0.341)	621 (0.583)	1.11 (0.92-1.35)	0.26	1.09 (0.82-1.44)	0.54
Mixed Eastern Western Polynesian	4(0.191)	7(0.333)	10(0.476)	27 (0.643)	3(0.167)	9(0.500)	6 (0.333)	21 (0.583)	1.25 (0.53-2.96)	0.61	9.15 (0.96-87.65)	0.05
Western Polynesian	18(0.063)	129(0.453)	138(0.484)	405 (0.711)	24(0.145)	66(0.400)	75(0.455)	216 (0.655)	1.30 (0.97-1.75)	0.08	1.21 (0.82-1.77)	0.33
Ngati Porou Hauora	25(0.120)	93(0.447)	78(0.375)	249 (0.635)	38(0.179)	101(0.476)	68 (0.321)	237 (0.572)	1.30 (0.98-1.73)	0.07	1.25 (0.83-1.88)	0.29

Adjusted against sex, age, body mass index, sugar-sweetened beverage (SSB) consumption (number of drinks/day) and alcohol consumption (g/week). CI, confidence interval; OR, odds ratio.

**Table 2:** **Association of *rs2544390 *and serum urate** (**mmol**/**L**)

**Ancestral group**	**Number**	**Unadjusted β-coeff(95% CI)**	***P-value***	**Adjusted β-coeff (95% CI)**	***P*-value**
All European Caucasian non-gout	7451	-0.00099 (-0.0029 to 0.0049)	0.62	-0.00029 (-0.0041 to 0.0035)	0.88
NZ Māori and Pacific Island non-gout	775	0.0068 (-0.0095 to 0.0232)	0.41	-0.0015 (-0.016 to 0.013)	0.84

Adjusted against sex, age, body mass index, sugar-sweetened beverage consumption (number of drinks/day) and study data set. The sample sets of Māori and Pacific Island ancestry are adjusted for the number of self-reported Māori and/or Pacific Island grandparents. CI, confidence interval; NZ, New Zealand.

Alcohol intake was positively associated with serum urate in unstratified European Caucasian and Māori and Pacific Island control sample sets (Table 3 and Additional file 1: Table S2; adjusted β = 0.00007 mmol/L, *P* = 1.98x10^-9^ and β = 0.00008, *P* = 0.014 respectively). No significant association of serum urate with alcohol consumption was observed in gout sample sets of both ancestries (Table 3). However, we note that this analysis would be compromised by urate-lowering therapy. We did not adjust for this, given the lack of data on adherence to medication.

**Table 3:** Association of alcohol intake (g/week) with serum urate (mmol/L)

**Study group**	**β (95% CI)**	***P*-value**
All European Caucasian non-gout	0.00007 (0.00004 to 0.00009)	1.98E-09
NZ Māori and Pacific Island non-gout	0.00008 (0.00001 to 0.00014)	0.014
NZ European Caucasian gout	0.000007 (-0.00012 to 0.00013)	0.91
NZ Māori and Pacific Island gout	0.00007 (-0.00001 to 0.00015)	0.10

Adjusted against sex, age, body mass index, sugar-sweetened beverage consumption (number of drinks/day) and study data set. The sample sets of Māori and Pacific Island ancestry are adjusted for the number of self-reported Māori and/or Pacific Island grandparents. CI, confidence interval; NZ, New Zealand.

Alcohol intake, when analyzed as a continuous variable (g/week), was positively associated with risk of gout in the Māori and Pacific but not NZ European Caucasian sample sets (Table 4 and Additional file 1: Table S3; adjusted OR = 1.002, *P* = 0.004 and OR = 1.001, *P* = 0.26, respectively). Any alcohol intake, when analyzed as a dichotomized variable, was associated with a 36% increase in risk of gout in Māori and Pacific Island (OR = 1.36; *P* = 0.03), with no increase in gout risk observed in the NZ European Caucasian sample set (OR = 0.96; *P* = 0.86) (Table 4 and Additional file 1: Table S3). Examining alcohol types revealed evidence for an association of beer consumption with risk of gout in European Caucasian and Māori and Pacific Island groups and spirit consumption with risk of gout in European Caucasian (Table 4). There was evidence for consumption of wine associating with a reduced risk of gout in European Caucasian (Table 4).

**Table 4:** Association of alcohol intake with gout

	**Alcohol consumption as continuous variable (g/week)**				
**Alcohol group**	**Obs.**	**Unadjusted OR (95% CI)**	***P***	**Adjusted OR(95% CI)**	***P***
All alcohol types					
NZ European Caucasian	837	1.000 (0.999 to 1.002)	0.74	1.001 (0.999 to 1.004)	0.26
NZ Māori and Pacific Island	1,799	1.001 (0.999 to 1.002)	0.07	1.002 (1.001 to 1.003)	0.004
Beer					
NZ European Caucasian	837	1.002 (0.999 to 1.004)	0.11	1.003 (1.000 to 1.006)	0.03
NZ Māori and Pacific Island	1,799	1.002 (1.001 to 1.003)	0.002	1.003 (1.001 to 1.004)	0.002
Wine					
NZ European Caucasian	837	0.998 (0.996 to 1.000)	0.10	0.999 (0.996 to 1.001)	0.33
NZ Māori and Pacific Island	1,799	0.998 (0.994 to 1.002)	0.28	1.000 (0.996 to 1.005)	0.84
Spirits					
NZ European Caucasian	837	1.002 (0.999 to 1.006)	0.21	1.005 (1.000 to 1.011)	0.05
NZ Māori and Pacific Island	1,799	0.999 (0.997 to 1.001)	0.40	1.001 (0.998 to 1.005)	0.54
	**Alcohol consumption as dichotomized variable (No alcohol versus any alcohol intake)**				
All alcohol types					
NZ European Caucasian	837	0.79 (0.54 to 1.15)	0.22	0.96 (0.56 to 1.62)	0.86
NZ Māori and Pacific Island	1,799	0.98 (0.80 to 1.19)	0.83	1.36 (1.03 to 1.79)	0.03
Beer					
NZ European Caucasian	837	1.26 (0.93 to 1.69)	0.13	1.32 (0.88 to 1.98)	0.17
NZ Māori and Pacific Island	1,799	1.55 (1.26 to 1.90)	2.4 x 10^-5^	1.37 (1.03 to 1.81)	0.03
Wine					
NZ European Caucasian	837	0.60 (0.45 to 0.81)	0.001	0.66 (0.46 to 0.95)	0.03
NZ Māori and Pacific Island	1,799	0.66 (0.48 to 0.90)	0.009	1.44 (0.93 to 2.23)	0.11
Spirits					
NZ European Caucasian	837	1.22 (0.86 to 1.75)	0.27	1.59 (1.02 to 2.48)	0.04
NZ Māori and Pacific Island	1,799	0.70 (0.52 to 0.94)	0.017	1.30 (0.86 to 1.94)	0.21

Adjusted against sex, age, body mass index, sugar-sweetened beverage consumption (number of drinks/day) and study data set. The sample sets of Māori and Pacific Island ancestry are adjusted for the number of self-reported Māori and/or Pacific Island grandparents. CI, confidence interval, NZ, New Zealand, Obs = Number of observations; OR, odds ratio.

We then tested for interaction between alcohol consumption and *rs2544390* genotype (Table 5). Evidence for non-additive interaction was observed in the combined Māori and Pacific Island sample set when alcohol consumption was analyzed either as a continuous (*P*_Adjusted_ = 0.001) or dichotomized variable (*P*_Adjusted_ = 0.001) but not in European Caucasian. To visualize the nature of the interaction between alcohol consumption and *rs2544390*, adjusted ORs were calculated in four groups with T-allele negative and no alcohol consumption being the referent group (Table 6 and Additional file 1: Table S4). Within the Māori and Pacific Island T-allele negative group there was a 4.18-fold increase in risk with any exposure to alcohol (*P* = 6.6 × 10^-5^) compared to a 1.14-fold increase within the T-allele positive group (*P* = 0.40). Within the different alcohol categories for the Māori and Pacific Island group there was a significant interaction term for risk of gout for beer consumption (dichotomized variable; OR = 0.43 (0.21 to 0.90), *P*_Adjusted_ = 0.026) but not for wine (dichotomized variable; OR = 0.68 (0.23 to 1.95), *P*_Adjusted_ = 0.47) or spirit (dichotomized variable; OR = 0.50 (0.18 to 1.34), *P*_Adjusted_ = 0.18) consumption.

**Table 5:** Interaction terms between alcohol intake and rs2544390 genotype for gout risk

**Alcohol consumption as continuous variable**			
	**Obs.**	**OR (95% CI)**	***P***
NZ European Caucasian	837	0.996 (0.991 to 1.002)	0.18
NZ Māori and Pacific Island	1,799	0.992 (0.987 to 0.997)	0.001
**Alcohol consumption as dichotomized variable (No alcohol (reference) versus any alcohol intake)**			
NZ European Caucasian	837	0.45 (0.15 to 1.34)	0.15
NZ Māori and Pacific Island	1,799	0.27 (0.13 to 0.57)	0.001

Adjusted against sex, age, body mass index, sugar-sweetened beverage consumption (number of drinks/day) and study data set. The sample sets of Māori and Pacific Island ancestry are adjusted for the number of self-reported Māori and/or Pacific Island grandparents. CI, confidence interval; NZ, New Zealand; Obs = Number of observations; OR, odds ratio.

**Table 6:** Alcohol intake and gout association results for groups stratified by rs2544390 genotype

**Study group**	**No alcohol**		**Any alcohol intake**	
	**OR (95% CI)**	***P***	**OR (95% CI)**	***P***
NZ European Caucasian				
T-	1.00	1	1.57 (0.68 to 3.63)	0.29
T+	1.45 (0.58 to 3.61)	0.43	1.02 (0.48 to 2.19)	0.95
NZ Māori and Pacific Island				
T-	1.00	1	4.18 (2.07 to 8.44)	6.61 x 10^-5^
T+	2.68 (1.54 to 4.66)	4.83 x 10^-4^	3.01 (1.72 to 5.28)	1.13 x 10^-4^

Adjusted against age, sex, body mass index, sugar-sweetened beverage consumption (number of drinks/day) and study data set. The sample sets of Māori and Pacific Island ancestry are adjusted for the number of self-reported Māori and/or Pacific Island grandparents. CI, confidence interval; NZ, New Zealand; OR, odds ratio.

## Discussion

Our study reports association of the T allele of *LRP2rs2544390* with gout risk in NZ Māori and Pacific Island samples (OR = 1.20, *P* = 0.009; Figure 1A). This observation is consistent with the reported association with increased serum urate and gout risk (OR = 1.32) of this allele in Japanese sample sets [5,6], supporting the conclusion that *LRP2 rs2544390* is a genuine risk factor for increased serum urate and gout in South East Asia and Oceania. In contrast, there was marginal evidence for association of *rs2544390* with gout risk in European Caucasian, but in an opposing direction (OR = 0.79, *P*_Unadjusted_ = 0.02). Heterogeneity (allelic and genetic) is also evident in gene associations with gout between among South East Asian, Polynesian and European Caucasian at *SLC2A9* and *ABCG2* (reviewed in [20]). If the association in European Caucasian were to be confirmed this would allow the inference that *rs2544390* is not causal. It could also be inferred that haplotypic structure would substantially differ at this locus in the two ancestral groupings, aiding in fine-mapping the causal variant. With respect to the location of the putative causal variant, in European Caucasian *rs2544390* is located (using 1,000 Genomes data) in a 40-kilobase haplotype block (SNPs with intermarker LD r^2^ >0.8) from 170.165 to 170.205 Mb on chromosome 2 wholly within *LRP2*; however, in Japanese the block is smaller at only 4-kilobases (170.201 to 170.205 Mb), spanning exons 84 and 85. This information would substantially reduce the amount of genome needing to be searched in fine-mapping the etiological variant.

There was a positive association between alcohol intake and serum urate in Māori and Pacific Island and European Caucasian control sample sets (Table 3) and between alcohol intake and risk of gout in Māori and Pacific Island only (Table 4). With respect to Māori and Pacific Island our data can be compared with that of Evans *et al*. [21] who observed that serum urate increases with reported alcohol intake in men of all ages of Polynesian ancestry. They also observed a higher prevalence of gout in people with high alcohol consumption. We confirm the relationship between alcohol intake, serum urate and risk of gout in people of Māori and Pacific Island ancestry, in a larger sample set and using contemporary statistical techniques that are able to adjust for potential measured confounders. As has previously been documented in European Caucasian [10], beer was more strongly associated with risk of gout than spirits in the Māori and Pacific Island samples, with no evidence for an association of wine consumption with risk of gout. In the NZ European Caucasian samples, the evidence for an association with risk of gout was strongest with beer and spirits consumption. However, in contrast to the Choi *et al*. study [10], we observed a protective relationship between wine consumption and risk of gout. Why this is the case is unclear, with confounding by other dietary behaviors a possible explanation. With respect to total alcohol consumption and gout risk in European Caucasian, the small NZ control sample size (n = 282) likely did not provide the power to detect a significant relationship. When alcohol intake was analyzed as a continuous variable, the direction and magnitude of the effect was similar in NZ European Caucasian to that in the Māori and Pacific Island sample set (Table 4).

There was evidence of non-additive interaction between rs2544390 genotype and alcohol consumption (Table 5; *P*_Interaction_ = 0.001) in the combined Māori and Pacific Island sample set in determining the risk of gout. The interaction was manifest by the normally protective genotype (CC) at rs2544390 conferring a significantly elevated risk of gout in alcohol drinkers compared to non-drinkers (Table 6). If this interaction is proven robust by replication, understanding of the biological mechanism may generate new knowledge on the role of alcohol in the etiology of gout, with the current paradigm dictating that alcohol increases urate by generation of purines in the bloodstream (mostly by hepatic metabolism). Alcohol exposure appears to over-ride the protective effect of the CC genotype. However, this observation is not consistent with Hamajima *et al*. [15]. In this study, they observed association of the TT genotype with the highest risk of hyperuricemia in male subjects consuming ≥5 alcoholic drinks per week (OR = 3.30). We undertook a similar analysis in men in the combined Māori and Pacific Island non-gout sample set (Additional file 1: Table S5). In our analysis the CC genotype was associated with the highest risk of hyperuricemia in those drinking ≥100 g of alcohol per week (OR = 4.48). Also, using the Hamajima *et al*. [15] data, we produced a similar analysis in men to that in Table 6 (Additional file 1: Table S5). The CC genotype group in the NZ Māori and Pacific Island sample had the highest risk for gout with alcohol exposure (OR = 3.96) whereas the CC genotype group exhibited neutral risk for hyperuricemia in the Japanese sample set (OR = 0.99). Thus, while the Hamajima *et al*. and our results both report non-additive interaction between alcohol consumption and *rs2544390* in hyperuricemia and gout, respectively, homozygosity for different alleles is associated with the highest risk upon exposure to alcohol. Given the consistency in *rs2544390* association with serum urate and gout risk between Māori and Pacific Island, and Japanese [5] (risk allele T), there is no obvious explanation for this discrepancy. Noting that there was no main effect association of *rs2544390* with serum urate in either European or Māori and Pacific Island control sample sets (Table 2), it is possible that the *rs2544390* association with gout and the alcohol-genotype interaction influencing gout risk in Polynesians is by a mechanism separate to the control of serum urate. It will be very important to observe non-additive interaction(s) between alcohol consumption and *rs2544390* in control of serum urate and gout in other datasets of South East Asian and Oceanic origin.

## Conclusions

The *LRP2rs2544390* SNP is associated with gout in people of Māori and Pacific ancestry, in a direction consistent with the previously reported effect on serum urate in Japanese. There is evidence for an opposing effect in European Caucasians, although this remains to be replicated. The genetic discrimination in risk of gout mediated by *rs2544390* is ablated by alcohol consumption in the NZ Māori and Pacific population. Further exploration of the mechanism underlying this interaction should generate new knowledge on the role of alcohol in gout, in addition to strengthening the evidence base for reduction of alcohol consumption in the management of gout.

## Abbreviations

ARIC: Atherosclerosis risk in communities; BMI: Body mass index; CEU: Centre d’Etude du Polymorphisme Humain from Utah; CI: Confidence interval; EP: Eastern Polynesian; FHS: Framingham heart study; LRP2: Lipoprotein receptor-related protein 2; NPH: Ngati Porou Hauora; OR: Odds ratio; SNP: Single nucleotide polymorphism; WP: Western Polynesian.

## Competing interests

The authors declare that they have no competing interests.

## Authors’ contributions

HR and TRM helped to design the study, oversee its execution, and prepare the manuscript. JEH-M, JHH, CF, ND, PBJ, DHNW and LKS helped to provide clinical recruitment and prepare the manuscript. AP-G and RT helped to collect data and prepare the manuscript. All authors read and approved the final manuscript.

## Supplementary Material

Additional file 1: Table S1 Demographic and clinical characteristics of study participants. **Table S2**: Association analysis of alcohol intake (g/week) with serum urate (mmol/L). **Table S3**: Association analysis of alcohol intake (g/week) with risk of gout. **Table S4**: Alcohol intake and gout risk for genotypically stratified groups in individual sample sets. **Table S5**: Top: Risk for hyperuricemia (serum urate ≥0.41mmol/L) among Japanese (gout and non-gout) and NZ Māori and Pacific Island (non-gout) males according to alcohol consumption and *rs2544390* genotype categories. Bottom: Risk for hyperuricemia amongst Japanese (gout and non-gout) males and for gout amongst NZ Māori and Pacific Island males according to alcohol consumption and *rs2544390* genotype categories. Click here for file
