# Supplementary material for: Association of the lipoprotein receptor-related protein 2 gene with gout and non-additive interaction with alcohol consumption
Source: Arthritis Res Ther. 2013 Nov 4;15(6):R177. doi: 10.1186/ar4366 (PMC3979038; doi:10.1186/ar4366)
Supplement: Additional file 1: Table S1 — Demographic and clinical characteristics of study participants. Table S2: Association analysis of alcohol intake (g/week) with serum urate (mmol/L). Table S3: Association analysis of alcohol intake (g/week) with risk of gout. Table S4: Alcohol intake and gout risk for genotypically stratified groups in individual sample sets. Table S5: Top: Risk for hyperuricemia (serum urate ≥0.41mmol/L) among Japanese (gout and non-gout) and NZ Māori and Pacific Island (non-gout) males according to alcohol consumption and rs2544390 genotype categories. Bottom: Risk for hyperuricemia amongst Japanese (gout and non-gout) males and for gout amongst NZ Māori and Pacific Island males according to alcohol consumption and rs2544390 genotype categories. [file ar4366-S1.doc]

Table S1: Demographic and clinical characteristics of study participants

|  | Eastern Polynesian | | Western Polynesian and Eastern/Western Polynesian | | Ngati Porou Hauora | | NZ European Caucasian | | ARIC | FHS |
| --- | --- | --- | --- | --- | --- | --- | --- | --- | --- | --- |
|  | Gout | Non-gout | Gout | Non-gout | Gout | Non-gout | Gout | Non-gout | Non-gout | Non-gout |
|  | 374 | 533 | 306 | 183 | 196 | 207 | 555 | 282 | 4144 | 3047 |
| Male Sex, n(%) | 274 (76.11) | 196 (36.91) | 286 (93.46) | 107 (59.12) | 160 (81.63) | 106 (51.21) | 445 (80.62) | 176 (62.63) | 1844 (44.50) | 1403 (46.05) |
| Age, years | 55.78 ± 12.47 | 43.95 ± 14.92 | 46.01 ± 11.76 | 38.87 ± 12.63 | 57.85 ± 13.11 | 42.68 ± 14.66 | 62.55 ± 13.82 | 50.13 ± 16.37 | 52.28 ± 5.51 | 40.06 ± 8.85 |
| Body mass index, kg/m2 | 35.04 ± 7.46 | 32.28 ± 7.56 | 37.63 ± 8.74 | 35.18 ± 7.14 | 36.21 ± 7.83 | 30.64 ± 5.94 | 30.24 ± 5.48 | 27.65 ± 6.52 | 26.62 ± 4.40 | 26.73 ± 5.38 |
| Serum urate, mmol/l | 0.416 ± 0.111 | 0.362 ± 0.084 | 0.467 ± 0.108 | 0.372 ± 0.084 | 0.445 ± 0.114 | 0.360 ± 0.088 | 0.396 ± 0.106 | 0.311 ± 0.078 | 0.329 ± 0.078 | 0.308 ± 0.089 |
| % Heart problems (% reported^1^) | 33.63 (89.84) | 7.54 (97.00) | 15.61 (98.37) | 2.25 (97.27) | 34.97 (93.37) | 10.78 (98.55) | 37.93 (99.28) | 6.12 (98.58) | 1.13 (99.81) | 1.64 (99.57) |
| % Kidney problems, (% reported) | 23.49 (88.77) | 3.49 (96.81) | 15.54 (96.73) | 1.14 (96.17) | 12.22 (91.84) | 1.69 (85.51) | 21.21 (98.56) | 2.53 (98.23) | 00 (99.71) | 00 (99.74) |
| % Hypertension (% reported) | 60.60 (89.57) | 20.78 (96.62) | 44.82 (97.71) | 13.81 (98.91) | 64.86 (94.39) | 17.73 (98.07) | 49.45 (99.10) | 14.80 (98.23) | 4.03 (99.69) | 8.96 (99.97) |
| % Diabetes (% reported) | 29.52 (88.77) | 13.96 (98.12) | 15.33 (98.04) | 10.56 (98.36) | 35.33 (93.88) | 6.44 (97.58) | 14.18 (99.10) | 3.99 (97.87) | 2.26 (99.83) | 1.32 (99.57) |
| Sugary drink consumption (servings/day) | 1.58 ± 1.98 | 1.48 ± 1.88 | 2.61 ± 2.37 | 2.10 ± 1.77 | 1.04 ± 2.00 | 1.49 ± 2.01 | 0.96 ± 1.40 | 0.89 ± 1.32 | 0.61 ± 0.60 | _ |
| Alcohol consumption (g/week) | 55.41 ± 125.93 | 42.56 ± 82.92 | 61.46 ± 123.13 | 38.2 ± 77.28 | 56.33 ± 93.77 | 68.03 ± 109.32 | 77.31 ± 103.83 | 74.17 ± 87.02 | 44.87 ± 88.45 | 65.4 ± 87.48 |
| % Self reported diuretic use (% reported) | 25.00 (75.94) | 5.00 (52.53) | 19.64 (89.87) | 3.14 (86.88) | 5.93 (68.88) | 9.49 (76.33) | 27.03 (86.67) | 2.65 (53.55) |  |  |
| % Allopurinol treatment (% reported) | 74.11 (89.84) | _ | 75.33 (99.67) | _ | 77.14 (89.29) | _ | 73.95 (98.92) | _ | _ | _ |
| Gout attacks per year | 8.24 ± 23.28 | _ | 11.81 ± 30.70 | _ | 2.94 ± 4.87 | _ | 6.45 ± 24.07 | _ | _ | _ |
| % Confirmed tophus (% reported) | 44.81 (48.93) | _ | 52.35 (48.69) | _ | 7.69 (13.27) | _ | 31.55 (57.12) | _ | _ | _ |
| % Suspected tophus (% reported) | 35.74 (85.29) | _ | 42.33 (98.04) | _ | 7.65 (93.37) | _ | 28.83 (98.74) | _ | _ | _ |

1 The percentage of the sample set from which information was available

Table S2: Association analysis of alcohol intake (g/week) with serum urate (mmol/L)

| Study Group | Number | Adjusted β-coeff [95%CI] | | *P* | |
| --- | --- | --- | --- | --- | --- |
| EP non-gout | 420 | 0.00004[-0.00006-0.00014] | 0.43 | |  |
| WP non-gout | 136 | (-)0.00001[-0.00017-0.00015] | 0.92 | |  |
| EP/WP non-gout | 16 | 0.0011[0.0005-0.0018] | 0.004 | |  |
| NPH non-gout | 207 | 0.0001[0.00003-0.00023] | 0.011 | |  |
| EP gout | 202 | 0.0001[-0.00002-0.00024] | 0.09 | |  |
| WP gout | 190 | 0.00003[-0.00011-0.00017] | 0.68 | |  |
| EP/WP gout | 11 | (-)0.0003[-0.00086-0.00018] | 0.15 | |  |
| NPH gout | 196 | 0.0001[-0.0006-0.0003] | 0.21 | |  |

Adjusted against sex, age, BMI, SSB consumption (drinks/day) and the number of self-reported Māori and/or Pacific Island grandparents.

Table S3: Association analysis of alcohol intake (g/week) with risk of gout

|  | Alcohol Consumption as Continuous Variable | |  |  |  |
| --- | --- | --- | --- | --- | --- |
| Study Groups | Obs. | Unadjusted OR[95%CI] | *P* | Adjusted OR[95%CI] | *P* |
| *All alcohol* |  |  |  |  |  |
| Eastern Polynesian | 907 | 1.001 [0.999-1.003] | 0.11 | 1.003 [1.001-1.005] | 0.008 |
| Western Polynesian | 450 | 1.002 [0.999-1.004] | 0.12 | 1.001 [0.998-1.003] | 0.61 |
| Eastern/Western Polynesian | 39 | 1.011 [1.000-1.021] | 0.03 | 1.013 [0.995-1.031] | 0.15 |
| Ngati Porou Hauora | 403 | 0.999 [0.997-1.001] | 0.34 | 1.003 [1.000-1.006] | 0.045 |
| *Beer* |  |  |  |  |  |
| Eastern Polynesian | 907 | 1.003 [1.001-1.005] | 0.003 | 1.004 [1.001-1.006] | 0.003 |
| Western Polynesian | 450 | 1.005 [1.001-1.009] | 0.02 | 1.002 [0.998-1.007] | 0.32 |
| Eastern/Western Polynesian | 39 | 1.011 [0.998-1.024] | 0.10 | 1.015 [0.996-1.035] | 0.12 |
| Ngati Porou Hauora | 403 | 1.000 [0.998-1.002] | 0.99 | 1.003 [1.000-1.006] | 0.035 |
| *Wine* |  |  |  |  |  |
| Eastern Polynesian | 907 | 0.996 [0.990-1.002] | 0.20 | 1.000 [0.994-1.007] | 0.97 |
| Western Polynesian | 450 | 0.998 [0.992-1.004] | 0.45 | 0.998 [0.992-1.004] | 0.51 |
| Eastern/Western Polynesian | 39 | 1.018 [0.980-1.057] | 0.37 | 1.214 [0.866-1.700] | 0.26 |
| Ngati Porou Hauora | 403 | 0.992 [0.978-1.006] | 0.27 | 1.004 [0.986-1.022] | 0.65 |
| *Spirits* |  |  |  |  |  |
| Eastern Polynesian | 907 | 0.999 [0.996-1.003] | 0.60 | 1.002 [0.997-1.006] | 0.48 |
| Western Polynesian | 450 | 1.001 [0.997-1.005] | 0.76 | 0.999 [0.994-1.006] | 0.97 |
| Eastern/Western Polynesian | 39 | 1.009 [0.986-1.032] | 0.46 | 1.008 [0.975-1.042] | 0.66 |
| Ngati Porou Hauora | 403 | 0.987 [0.976-0.997] | 0.015 | 0.992 [0.978-1.006] | 0.25 |
|  | Alcohol Consumption as Dichotomized Variable (No alcohol vs. any alcohol intake) | | | |  |
| *All alcohol* |  |  |  |  |  |
| Eastern Polynesian | 907 | 0.96 [0.71-1.30] | 0.81 | 1.04 [0.70-1.56] | 0.85 |
| Western Polynesian | 450 | 1.25 [0.84-1.87] | 0.27 | 1.29 [0.79-2.09] | 0.31 |
| Eastern/Western Polynesian | 39 | 7.58 [1.74-33.09] | 0.007 | 8.24 [0.77-88.57] | 0.08 |
| Ngati Porou Hauora | 403 | 0.79 [0.53-1.18] | 0.25 | 1.59 [0.88-2.85] | 0.12 |
| *Beer* |  |  |  |  |  |
| Eastern Polynesian | 907 | 1.58 [1.17-2.14] | 0.003 | 1.19 [0.78-1.83] | 0.42 |
| Western Polynesian | 450 | 1.94 [1.23-3.06] | 0.005 | 1.24 [0.71-2.18] | 0.46 |
| Eastern/Western Polynesian | 39 | 24.00 [2.63-218.65] | 0.005 | 568.50 [0.80-404139] | 0.06 |
| Ngati Porou Hauora | 403 | 1.19 [0.78-1.78] | 0.39 | 1.99 [1.05-3.74] | 0.03 |
| *Wine* |  |  |  |  |  |
| Eastern Polynesian | 907 | 0.64 [0.42-1.00] | 0.05 | 1.15 [0.63-2.09] | 0.65 |
| Western Polynesian | 450 | 0.60 [0.31-1.18] | 0.14 | 1.04 [0.41-2.62] | 0.94 |
| Eastern/Western Polynesian | 39 | 4.00 [0.40-39.83] | 0.24 | 98.74 [0.00-1.36E+07] | 0.45 |
| Ngati Porou Hauora | 403 | 0.90 [0.42-1.93] | 0.79 | 2.70 [0.85-8.61] | 0.09 |
| *Spirits* |  |  |  |  |  |
| Eastern Polynesian | 907 | 0.75 [0.49-1.15] | 0.19 | 1.39 [0.78-2.47] | 0.27 |
| Western Polynesian | 450 | 1.05 [0.58-1.91] | 0.87 | 1.65 [0.75-3.62] | 0.21 |
| Eastern/Western Polynesian | 39 | 0.52 [0.08-3.54] | 0.50 | 0.96 [0.03-27.82] | 0.98 |
| Ngati Porou Hauora | 403 | 0.33 [0.16-0.69] | 0.003 | 0.45 [0.15-1.33] | 0.15 |

Adjusted against sex, age, BMI, SSB consumption (drinks/day) and the number of self-reported Māori and/or Pacific Island grandparents.

Table S4: Alcohol intake and gout risk for genotypically stratified groups in individual sample sets

| **Study Groups** | **No Alcohol** | | **Alcohol intake** | |
| --- | --- | --- | --- | --- |
|  | **OR [95%CI]** | ***P*** | **OR [95%CI]** | ***P*** |
| Eastern Polynesian |  |  |  |  |
| T- | 1 | 1 | 3.05 [1.15-8.12] | 0.03 |
| T+ | 1.90 [0.89-4.06] | 0.09 | 1.75 [0.79-3.88] | 0.17 |
| Western Polynesian |  |  |  |  |
| T- | 1 | 1 | 2.58 [0.53-12.51] | 0.24 |
| T+ | 3.29[1.02-10.62] | 0.05 | 3.48 [1.06-11.39] | 0.04 |
| EP/WP |  |  |  |  |
| T- | 1 | 1 | - | - |
| T+ | 1.2E+07 [1.61-9.54E+13] | 0.04 | 5.9E+08 [910.01-3.9E+14] | 0.003 |
| Ngati Porou Hauora |  |  |  |  |
| T- | 1 | 1 | 4.37 [0.99-19.27] | 0.051 |
| T+ | 2.09 [0.61-7.21] | 0.24 | 3.15 [0.95-10.47] | 0.061 |

Adjusted against sex, age, BMI, SSB consumption (drinks/day) and the number of self-reported Māori and/or Pacific Island grandparents.

Table S5. Top: Risk for hyperuricemia (serum urate ≥0.41mmol/L) among Japanese (gout and non-gout) and NZ Māori and Pacific Island (non-gout) males according to alcohol consumption and *rs2544390* genotype categories. Bottom: Risk for hyperuricemia amongst Japanese (gout and non-gout) males and for gout amongst NZ Māori and Pacific Island males according to alcohol consumption and *rs2544390* genotype categories.

| Equivalent to Hamajima et al Table 7 |  | Genotype | Alcohol exposure | Normal, n (%) | Hyperuricemia, n (%) | | OR, 95% CI |
| --- | --- | --- | --- | --- | --- | --- | --- |
| Hamajima et al. | | CC | No | 121 (79.1) | 32 (28.9) | | 1 |
|  | |  | <5 drinks/day | 162 (79.0) | 43 (21.0) | | 1.10 [0.65-1.86] |
|  | |  | ≥5 drinks/day | 220 (79.4) | 57 (28.6) | | 1.11 [0.67-1.40] |
|  | | CT | No | 227 (80.8) | 54 (19.2) | | 1 |
|  | |  | <5 drinks/day | 326 (69.7) | 142 (30.3) | | 1.55 [1.07-2.26] |
|  | |  | ≥5 drinks/day | 417 (70.0) | 179 (30.0) | | 1.75 [1.22-2.51] |
|  | | TT | No | 126 (85.1) | 22 (14.9) | | 1 |
|  | |  | <5 drinks/day | 156 (77.6) | 45 (22.4) | | 1.64 [0.93-2.90] |
|  | |  | ≥5 drinks/day | 163 (67.9) | 77 (32.1) | | 3.13 [1.80-5.43] |
| NZ Māori/Pacific Island^1^ | | CC | No | 16 (57.1) | 12 (42.9) | | 1 |
|  | |  | <100 g/week | 8 (72.7) | 3 (27.3) | | 0.48 [0.09-2.40] |
|  | |  | ≥100 g/week | 3 (25.0) | 9 (75.0) | | 4.48 [0.76-26.13] |
|  | | CT | No | 40 (57.1) | 30 (42.9) | | 1 |
|  | |  | <100 g/week | 18 (51.4) | 17 (48.6) | | 1.29 [0.53-3.13] |
|  | |  | ≥100 g/week | 24 (60.0) | 16 (40.0) | | 0.97 [0.41-2.26] |
|  | | TT | No | 25 (50.0) | 25 (50.0) | | 1 |
|  | |  | <100 g/week | 12 (42.9) | 16 (57.1) | | 1.56 [0.57-4.26] |
|  | |  | ≥100 g/week | 13 (50.0) | 13 (50.0) | | 1.20 [0.42-3.39] |
| Equivalent to Humaira et al Table 6 | | | No alcohol OR [95% CI] | *P* | Any alcohol OR [95% CI] | *P* | |
| Hamajima et al. | | T- | 1 | 1 | 0.99 [0.63-1.55] | | 0.96 |
|  | | T+ | 0.81 [0.51-1.29] | 0.38 | 1.58 [1.05-2.37] | | 0.027 |
| NZ Māori/Pacific Island^1^ | | T- | 1 | 1 | 3.96 [1.67-9.41] | | 0.002 |
|  | | T+ | 2.38 [1.20-4.73] | 0.013 | 2.78 [1.40-5.53] | | 0.004 |

1 NZ odds ratios adjusted against age, BMI, and number of self-reported Māori and/or Pacific Island grandparents.
